# Author Correction: Cross-reactive immunity against the SARS-CoV-2 Omicron variant is low in pediatric patients with prior COVID-19 or MIS-C

**DOI:** 10.1038/s41467-022-32572-x

**Published:** 2022-08-12

**Authors:** Juanjie Tang, Tanya Novak, Julian Hecker, Gabrielle Grubbs, Fatema Tuz Zahra, Lorenza Bellusci, Sara Pourhashemi, Janet Chou, Kristin Moffitt, Natasha B. Halasa, Stephanie P. Schwartz, Tracie C. Walker, Keiko M. Tarquinio, Matt S. Zinter, Mary A. Staat, Shira J. Gertz, Natalie Z. Cvijanovich, Jennifer E. Schuster, Laura L. Loftis, Bria M. Coates, Elizabeth H. Mack, Katherine Irby, Julie C. Fitzgerald, Courtney M. Rowan, Michele Kong, Heidi R. Flori, Aline B. Maddux, Steven L. Shein, Hillary Crandall, Janet R. Hume, Charlotte V. Hobbs, Adriana H. Tremoulet, Chisato Shimizu, Jane C. Burns, Sabrina R. Chen, Hye Kyung Moon, Christoph Lange, Adrienne G. Randolph, Surender Khurana

**Affiliations:** 1 Division of Viral Products, Center for Biologics Evaluation and Research (CBER), FDA, Silver Spring, MD 20993 USA; 2 Department of Anesthesiology, Critical Care and Pain Medicine, Boston Children’s Hospital, Boston, MA 02115 USA; 3 Department of Anesthesia, Harvard Medical School, Boston, MA 02115 USA; 4 Channing Division of Network Medicine, Department of Medicine, Brigham and Women’s Hospital and Harvard Medical School, Boston, MA 02115 USA; 5 Department of Pediatrics, Boston Children’s Hospital and Harvard Medical School, Boston, MA 02115 USA; 6 Division of Immunology, Boston Children’s Hospital, Harvard Medical School, Boston, MA 02115 USA; 7 Division of Infectious Diseases, Boston Children’s Hospital, Boston, MA 02115 USA; 8 Division of Pediatric Infectious Diseases, Department of Pediatrics, Vanderbilt University Medical Center, Nashville, TN 37232 USA; 9 Department of Pediatrics, University of North Carolina at Chapel Hill Children’s Hospital, Chapel Hill, NC 27514 USA; 10 Division of Critical Care Medicine, Department of Pediatrics, Emory University School of Medicine, Children’s Healthcare of Atlanta, Atlanta, GA 30322 USA; 11 Department of Pediatrics, Divisions of Critical Care and Bone Marrow Transplantation, University of California, San Francisco, San Francisco, CA 94158 USA; 12 Department of Pediatrics, University of Cincinnati, Division of Infectious Diseases, Cincinnati Children’s Hospital Medical Center, Cincinnati, OH 45229 USA; 13 Division of Pediatric Critical Care, Department of Pediatrics, Cooperman Barnabas Medical Center, Livingston, NJ 07039 USA; 14 Division of Critical Care Medicine, UCSF Benioff Children’s Hospital Oakland, Oakland, CA 94609 USA; 15 Division of Pediatric Infectious Diseases, Department of Pediatrics, Children’s Mercy Kansas City, Kansas City, MO 64108 USA; 16 Division of Critical Care Medicine, Department of Pediatrics, Baylor College of Medicine, Houston, TX 77030 USA; 17 Division of Critical Care Medicine, Department of Pediatrics, Northwestern University Feinberg School of Medicine, Ann & Robert H. Lurie Children’s Hospital of Chicago, Chicago, IL 60611 USA; 18 Division of Pediatric Critical Care Medicine, Medical University of South Carolina, Charleston, SC 29425 USA; 19 Section of Pediatric Critical Care, Department of Pediatrics, Arkansas Children’s Hospital, Little Rock, AR 72202 USA; 20 Division of Critical Care, Department of Anesthesiology and Critical Care, The University of Pennsylvania Perelman School of Medicine, Philadelphia, PA 19104 USA; 21 Division of Pediatric Critical Care Medicine, Department of Pediatrics, Indiana University School of Medicine, Riley Hospital for Children, Indianapolis, IN 46202 USA; 22 Division of Pediatric Critical Care Medicine, Department of Pediatrics, University of Alabama at Birmingham, Birmingham, AL 35233 USA; 23 Division of Pediatric Critical Care Medicine, Department of Pediatrics, Mott Children’s Hospital and University of Michigan, Ann Arbor, MI 48109 USA; 24 Department of Pediatrics, Section of Critical Care Medicine, University of Colorado School of Medicine and Children’s Hospital Colorado, Aurora, CO 80045 USA; 25 Division of Pediatric Critical Care Medicine, Rainbow Babies and Children’s Hospital, Cleveland, OH 44106 USA; 26 Division of Pediatric Critical Care, Department of Pediatrics, University of Utah, Salt Lake City, UT 84113 USA; 27 Division of Pediatric Critical Care, University of Minnesota Masonic Children’s Hospital, Minneapolis, MN 55454 USA; 28 Department of Pediatrics, Department of Microbiology, Division of Infectious Diseases, University of Mississippi Medical Center, Jackson, MS 39202 USA; 29 Kawasaki Disease Research Center, Rady Children’s Hospital and Department of Pediatrics, UCSD School of Medicine, La Jolla, CA 92093 USA; 30 Department of Biostatistics, Harvard T.H. Chan School of Public Health, Boston, MA 02115 USA

**Keywords:** Viral infection, SARS-CoV-2, Antibodies, Vaccines

Correction to: *Nature Communications* 10.1038/s41467-022-30649-1, published online 27 May 2022

The original version of the Supplementary Information associated with this Article omitted Supplementary Figs. S1 and S2. The HTML has been updated to include a corrected version of the Supplementary Information.

## Supplementary information

Supplementary Information

